# A 3-Arm, Randomized, Controlled Trial of Heat-Sensitive Moxibustion Therapy to Determine Superior Effect among Patients with Lumbar Disc Herniation

**DOI:** 10.1155/2014/154941

**Published:** 2014-07-24

**Authors:** Rixin Chen, Mingren Chen, Tongsheng Su, Meiqi Zhou, Jianhua Sun, Jun Xiong, Zhenhai Chi, Dingyi Xie, Bo Zhang

**Affiliations:** ^1^The Affiliated Hospital with Jiangxi University of TCM, No. 445 Bayi Avenue, Nanchang 330006, China; ^2^Shanxi TCM Hospital, Xian 710003, China; ^3^The First Affiliated Hospital with Anhui University of TCM, Hefei 230031, China; ^4^Jiangsu TCM Hospital, Nanjing 210029, China

## Abstract

Systematic reviews of moxibustion for LDH have identified ponderable evidence, especially for heat-sensitive moxibustion (HSM). Therefore, we designed and carried out the large sample trial to evaluate it. 456 patients were recruited from 4 centers in China and were randomly divided into three groups by the ratio of 1 : 1 : 1 to HSM (152) group, conventional moxibustion (152) group, and conventional drug plus acupuncture (152) group. Compared with usual care, there was a statistically significant reduction in mean M-JOA score at 2 weeks and 6 months for HSM (3.8 ± 2.6 versus 8.5 ± 2.9; 3.7 ± 2.2 versus 10.1 ± 2.9) and conventional moxibustion (7.9 ± 3.0 versus 8.5 ± 2.9; 8.9 ± 3.1 versus 10.1 ± 2.9). Compared with conventional moxibustion group, HSM group showed greater improvement in all the outcomes. The mean dose of moxibustion was 41.13 ± 5.26 (range 21–60) minutes in the HSM group. We found that HSM was more effective in treating patients with LDH, compared with conventional moxibustion and conventional drug plus acupuncture. This finding indicated that the application of moxibustion on the heat-sensitive points is a good moxibustion technique in treating disease.

## 1. Background

Therapies to strengthen the motor function and relieve low back pain are the most commonly recommended treatment for lumbar disc herniation (LDH), such as acupuncture and moxibustion [1]. They have the advantage better than other therapies (especially surgery) that they have no physical side-effects or adverse reactions [2, 3]. Moxibustion is a traditional oriental therapy that treats diseases through thermal stimulation of burning herbs, primarily* Artemisia vulgaris*, at specific acupuncture and moxibustion point on the skin [4]. Traditional Chinese medicine (TCM) considers LDH to be the result of an unbalanced state among interfunctioning organs or a block vital energy (called Qi) condition with characteristic blood symptoms [5]. A large number of clinical studies have shown positive results of moxibustion remedies on LDH [6]. And moxibustion therapy has been important treatment in China. In particular, moxibustion treatment is effective for functional limitation and pain symptom because it provides warm energy, expels Qi-blood stagnation, and enhances local blood circulation [7]. Experimental studies showed moxibustion had anti-inflammatory or immunomodulatory effects against chronic inflammatory conditions in humans [8].

For moxibustion therapy, many factors influenced the therapeutic effect. However, the first thing to think about is the selection of location for manipulating moxa [9]. Conventional moxibustion applied moxibustion on fix acupuncture points based on pattern differentiation. Different patients received treatments on the same acupuncture points. However, heat-sensitive moxibustion (HSM) selected location that received moxibustion differently [10]. Heat-sensitive moxibustion administered moxibustion on heat-sensitive acupuncture points, which are extremely sensitive to the heat stimulation of burning moxa [11]. By using such acupuncture points, it is easier for channel Qi to transmit and to allow a strong response to be produced by weak stimulation. Patients felt heat-sensitive sensation on these acupuncture points [12].

According to acupuncture point sensitized theory, there are two kinds of state in acupuncture points in human body: stimulated state and resting state. When people get sick, the acupoints on the body surface area are activated and sensitized. Our research found that the heat-sensitive phenomenon to acupuncture point or an area is a new type of reaction in a pathological state. The sensitive areas are susceptible to heat stimulation and called “heat-sensitive acupuncture points.” A feature of these areas is that these areas are specific or closely relevant to acupuncture points and produce the same clinical effect as “a small stimulation induces a large response.” This heat-sensitive acupuncture point is not only the pathological phenomenon reflection of the diseases but also an effective stimulating location with acupuncture and moxibustion. These heat-sensitized locations are not fixed, but may, during the progression of disease, dynamically change within a certain range centered on acupuncture points [13]. Our empirical evidence engaged us in formulating the following hypothesis: moxibustion at the heat-sensitive acupuncture points showed better efficacy than that at fixed acupuncture points.

However TCM theory in China agreed that the best place to apply moxibustion was on heat-sensitive acupuncture points, because using them led to better stimulation and transmission of channel Qi. When Qi arrives at one part of the body, it can treat the diseases nearby. In the part of* Miraculous pivot*,* the chapter of nine needles and twelve sources* said: “The key point of acupuncture is the arrival of Qi, it ensures therapeutic effect.” However, there is little high-quality clinical evidence of its effectiveness. Therefore, we designed and carried out the large sample trial to evaluate it.

The results of a recent meta-analysis of six randomized controlled trials (RCTs) on moxibustion for LDH manifested that heat-sensitive therapy presented a favorable effect on LDH symptom scores compared with that of the drug [RR = 1.91, 95% CI (1.01, 3.60)] [14]. However, because of the number of eligible RCTs and the high risk of bias in the assessment of the available RCTs, the evidence supporting this conclusion is limited. Therefore, this well-designed and big sample RCT was needed to establish the efficacy of heat-sensitive moxibustion for LDH.

## 2. Methods

### 2.1. Objective

The aim of this study is to assess the effectiveness of heat-sensitive moxibustion for treating LDH compared with conventional drug plus acupuncture as well as conventional moxibustion.

### 2.2. Sample Size

An effect size on the M-JOA was sought when comparing the heat-sensitive with conventional moxibustion. In our previous pilot study, the effective rate in heat-sensitive moxibustion group is 65% and 45% in the other groups. An allocation ratio of 1 : 1 : 1 was chosen in order to increase power to detect statistically significant differences between the three groups. With 90% power and a two-sided significance level of 5%, the required group sizes were 126. Allowing for 20% attrition, the total sample size required was 456 (i.e., groups of 152, 152, 152, resp.):

(1) $$\begin{array}{c} n=\frac{p_{1}\times \left(1-p_{1}\right)+p_{2}\times \left(1-p_{2}\right)}{{\left(p_{2}-p_{1}\right)}^{2}}\times f\left(\alpha ,\beta \right). \end{array}$$

### 2.3. Design

We performed a multicenter (four centers in China), randomized, assessor blinded, and positive controlled trial. Our trial was carried out in four hospitals in China, including the Affiliated Hospital of Jiangxi University of Traditional Chinese Medicine (TCM) in Nanchang, the first Affiliated Hospital of Anhui University of TCM in Hefei, Jiangsu TCM Hospital in Nanjing, and Shanxi TCM Hospital in Xian. Patients were recruited through hospital-based recruitment and newspaper advertisements. After a baseline phase of one week, we used a central randomization system (random list generated with computer telephone integration by the statistician from China Academy of Chinese Medical Sciences) to randomize patients [15]. All study participants provided written, informed consent, and the study conformed to common guidelines for clinical trials (Declaration of Helsinki, ICH-GCP, including certification by external audit). The evaluation of participants and the analysis of the results were performed by professionals blinded to the group allocation.

### 2.4. Participants

#### 2.4.1. Recruitment

Patients were recruited in China from December 30, 2011, to January 30, 2013. Informed consent was obtained from each subject, and the Ethics Committee of Affiliated Hospital of Jiangxi Institute of Traditional Chinese Medicine, China, approved the study protocol, authorization number: 2008(11).

#### 2.4.2. Inclusion Criteria

Inclusion criteria were a diagnosis of LDH according to the guiding principle of clinical research on new drugs (GPCRND) [16], at least 10 scores in M-JOA in the baseline period, age 18–65 years, pain occurring in lower back and radiating to the lower limb, completed baseline LDH diary, and written informed consent. Meanwhile, heat-sensitive acupuncture points were found in the triangle region formed with bilateral Dachangshu (BL25) and Yaoshu (Du2) of patients (Dachangshu-Yaoshu-contralateral Dachangshu intraregion).

#### 2.4.3. Exclusion Criteria

Main exclusion criteria were patients with serious life-threatening disease, such as disease of the heart and brain, blood, vessels, liver, kidney, and hematopoietic system, pregnant or lactating female, and psychotic patients. We also excluded patients with a single nerve palsy, or cauda equina nerve palsy, manifested as muscle paralysis or having rectum or bladder problems; complicated with lumbar spinal canal stenosis and space-occupying lesions or for other reasons; complicated with lumbar spine tumors, infections, tuberculosis; complicated with moxibustion syncope and unwilling to be treated with moxibustion; patients do not sign informed consent.

### 2.5. Study Interventions

We developed the study interventions in a consensus process with China acupuncture experts and societies. Physicians trained and experienced (at least five years) in acupuncture delivered the interventions. All treatment regimens were standardized between four centers practitioners via video, hands-on training, and internet workshops. In the moxibustion groups, 22 mm (diameter) × 120 mm (length) moxa-sticks (Jiangxi Traditional Chinese Medicine Hospital, China) were used. The patient was usually in the comfortable supine position for treatment, with 24°C to 30°C temperature in the room.

#### 2.5.1. Heat-Sensitive Moxibustion Group

For the heat-sensitive moxibustion group, moxibustion treatment was defined as burning a moxa-stick with the patient lying on his or her back. The moxa-sticks were lit by the therapist and held over the region among two Dachangshu (BL25) and Yaoshu (Du 2) of patients. The moxa-stick suspended at an approximate distance of 3 cm was used to search for acupuncture points showing the heatsensitisation phenomenon. The following patients sensation suggested the special heat-sensitization acupuncture points: heat penetration, patients reporting heat penetrating from the skin into subcutaneous tissues; heat expansion, heat expanding away from the stimulation site to surrounding cutaneous and subcutaneous tissues; heat transmission, patients perceiving a stream of heat conducting in certain directions or perceiving heat in some body regions or into the joint cavity; nonthermal sensations, instead of thermal sensations, some patients perceiving aching, heaviness, pain, numbness, pressure, or cold in local or distant locations of stimulation. When such an acupuncture point was found, the therapists marked the point. We tried our best to seek all the special acupuncture points in each patient by the repeated manipulation.

The therapists began to treat patients from the most heat-sensitive intensity acupoint. Treatment sessions ended when patients felt the acupoint heat-sensitization phenomenon had disappeared. Generally speaking, one point was selected each time. One point was treated 30~60 minutes. Patients received the treatment for two times daily in the first four days and for one time daily in remaining ten days. The whole treatment contained 18 sessions over 14 days.

#### 2.5.2. Conventional Moxibustion Group

A licensed doctor performed fixed acupuncture point moxibustion. Common practices were similar to the first group. The different manipulation was that the therapists carry out warming moxibustion in traditional acupuncture point, selecting Dachangshu (BL25), Weizhong (BL40), and A-shi Xue. One point is treated 15 minutes a time. The whole process of moxibustion took about 45 minutes for each session. Patients usually felt local warmth without burning pain and might experience mild hyperemia in the local region. The sensation of acupuncture point heat-sensitization phenomenon was not pursued and not avoided in the treatment. Patients received the treatment for two times daily in the first four days and for one time daily in the remaining ten days. The whole treatment contained 18 sessions over 14 days.

#### 2.5.3. Conventional Drug Plus Acupuncture Group

For conventional drug, patients received the 20% mannitol (250 mL, intravenously) and Voltaren tablets (75 mg, 2 times a day) in the first 3 days. Voltaren tablets were continued to be used in the subsequent 11 days. At the same time, acupuncture needles were used and acupuncture points selected from Bladder Meridian of Foot-Taiyang and Gallbladder Meridian of Foot-Shaoyang. Acupoints included Dachangshu (BL25), Yaojiaji (EX-B2), Huantiao (GB30), Weizhong (BL40), Yanglingquan (GB34), Xuanzhong (GB39), and Qiuxu (GB40). We selected bilateral acupoints located in waist and ipsilateral acupoints located in lower limbs. Needles remained in acupuncture point for 30 minutes. Patients received the acupuncture needle treatment one time/day in two weeks for a total of 14 sessions over 14 days.

### 2.6. Outcome Measures

Our primary outcome measure was the M-JOA. The JOA has proposed a series of criteria to define patient response in the context of clinical trials of LDH. M-JOA scale is a modified edition of JOA Back Pain Evaluation Questionnaire. According to these criteria, a patient with LDH is assessed for pain, the ability to conduct daily life and work, functional impairment, and particular clinical examinations. M-JOA scores range from 0 to 24, with LDH considered mild (0–9), moderate (10–20), or severe (21 and above). The M-JOA was used as a preference-based measure of health outcome. All patients were assessed before randomization (baseline phase), 2 weeks after randomization, and 6 months after the last treatment. This trial also recorded adverse effects reported by patients during treatment.

We ensured assessor blinding in this trial. Patients were informed not to tell outcome assessors the treatment they received. The outcome assessor was not involved in treatment administration.

### 2.7. Statistical Methods

Data were analysed on an intention-to-treat (ITT) basis including all randomised participants with at least one measurable outcome report. The statistician conducting the analyses remained blinded to treatment groups. All analyses were conducted using SPSS 11.5. The groups were compared on 2 weeks, with *t*-tests used to assess changes between baseline and 2 weeks within each arm. ANOVA was used to compare these changes among the three treatment arms of the trial. Where a significance difference was found among the three groups, pair-wise tests were used to determine specifically which groups differed significantly. Student-Newman-Keuls was used for pairwise comparison. All adverse reactions manifested were listed with detailed explanations. A significance level of 5% was used in all analyses.

## 3. Results

### 3.1. Population and Baseline

**Figure 1:**
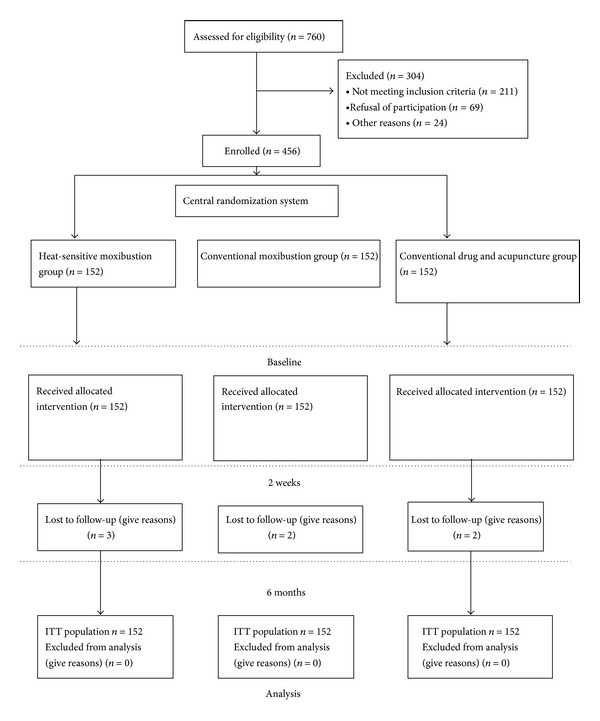
A flowchart of the study process.

Participants were recruited from outpatients and inpatients in the four study centers. Patient flow in the trial was presented in Figure 1. After screening 760 patients, 456 were randomly assigned to treatment. 304 could not be included in the study, mainly because they did not meet all eligibility criteria. After six months, 7 patients missed. Reasons for missing follow-up data were not contactable. Participants had a mean age of 46.3 years, and 52.4% were female.  Table 1 presented the history of LDH of the subjects. The mean M-JOA score was 17.6. Baseline patient characteristics were balanced between the trial arms. There was no difference in attrition rate among the groups at 6-month follow-up (*P* > 0.05, Fisher exact test).

#### 3.1.1. Total M-JOA Score

There was a significant reduction in mean M-JOA score from baseline in all three groups (*P* < 0.01). ANOVA test showed significant difference in the three groups at both time points. Mixed-effects model analysis (*q*-test) showed that subjects in the heat-sensitive moxibustion group had significantly greater reduction in M-JOA scores than those in conventional moxibustion group or conventional drug plus acupuncture group at 2 weeks and 6 months; however, there was no significant difference between conventional moxibustion and conventional drug plus acupuncture at both time points (Table 2).

### 3.2. Moxibustion Time in the Heat-Sensitive Moxibustion Group

Different from the conventional moxibustion group, moxibustion dose was individual in the heat-sensitive moxibustion group. According to the record of individual moxibustion time, the dose differed in terms of patients' conditions and moxibustion sensation, which had been measured about 21~60 minutes in the treatment of LDH. The range of mean moxibustion dose was about 41.13 ± 5.26 minutes in the conventional moxibustion group. We used a linear correlation to measure the strength of a relationship between change in M-JOA score and stimulation duration in the conventional moxibustion group. The Pearson coefficient *r* = 0.0006, showing a poor correlation between the two values.

### 3.3. Safety

No adverse events were reported in the 456 participants.

## 4. Discussions

The heat-sensitive moxibustion intervention tested in this study was significantly more effective than conventional moxibustion treatment and significantly more effective than the conventional drug plus acupuncture intervention in patients with LDH. No serious cases of adverse reactions related to treatment were reported. This study had a clear and practical research question with an appropriate trial design, namely, a pragmatic randomized controlled trial, which modelled closely what would happen if patient refers to moxibustion. Compared with available studies of moxibustion for LDH, which included a maximum amount of 120 patients [17–19], our study has a much larger sample size. Other advantages included adherence to current guidelines for acupuncture trials, strictly concealed central randomization, blinded evaluation of statistics and measurement, interventions based on expert consensus provided by qualified and experienced medical acupuncturists, and high follow-up rates. Trial physicians could not be blinded. It was not possible to blind the conventional drug plus acupuncture patients. Therefore, the large and significant difference between HSM and conventional moxibustion and between HSM and conventional drug plus acupuncture could be due to performance bias and detection bias. The results of this study proved the superiority of heat-sensitive moxibustion in patients suffering from LDH. That is, selecting the heat-sensitive acupuncture point obtained therapeutic effect far better than moxibustion at acupuncture point of routine resting states. These heat-sensitive acupuncture points are not fixed, but may, during the progression of disease, dynamically change within a certain range centered on acupuncture points. Several types of heat-sensitization responses might appear alone or in combination. Patients become thermally sensitized to moxibustion stimulation at certain locations on the body, indicated by sensations of strong warmth or heat penetrating into the body (heat penetration), warmth spreading around the stimulation site (heat expansion), warmth conducting in certain directions and reaching some body regions or even internal organs remote from stimulation sites (heat transmission), or other nonthermal sensations [20]. These responses gradually disappear with disease recovery.

In summary, we have provided high-quality evidence that heat-sensitive moxibustion showed significant reduction in symptoms of LDH in the short and long term compared with other two treatments (conventional moxibustion, conventional acupuncture plus medicine). The importance of the therapeutic relationship providing heat-sensitive acupuncture point should not be underestimated in the moxibustion therapy. Therefore, the success of this project is more than providing the efficacy of heat-sensitive moxibustion as a treatment modality in patients with LDH. The findings will be helpful to provide better therapeutic options to enhance the efficacy of moxibustion and to perfect acupuncture point heat-sensitive theory.

## Figures and Tables

**Table 1:** Baseline characteristics of participants.

Items	Heat-sensitive moxibustion	Conventional moxibustion	Conventional drug plus acupuncture
	group	group	group
Age, mean (SD), years	45.5 (10.6)	47.3 (11.2)	46.6 (10.5)
Age, min~max, years	18~59	20~58	18~59
Age, >60 y, *n* (%)	9 (5.92)	10 (6.6)	9 (5.92)
Sex *n* (%)			
Female	78 (51.3)	80 (52.6)	81 (53.3)
Male	74 (48.7)	72 (47.4)	71 (46.7)
Duration of pain *n* (%)			
<1 m	32 (21.1)	30 (19.7)	30 (19.7)
2~6 m	40 (26.3)	42 (27.6)	43 (28.2)
7~12 m	40 (26.3)	33 (21.7)	31 (20.3)
1~5 y	33 (21.7)	38 (25.1)	40 (26.3)
>5 y	7 (4.6)	9 (5.9)	8 (5.2)
BMI, mean (SD), kg/m′	22.2 (3.3)	22.4 (3.1)	21.1 (4.0)
BMI, min~max, kg/m′	14.3~30.1	16.2~29.2	13.1~28.9
M-JOA score *n* (%)			
Severe	115 (75.6)	113 (73.4)	119 (78.3)
Moderate	37 (24.4)	39 (25.6)	33 (21.7)
M-JOA score, mean (SD)	18.6 (3.8)	17.5 (3.3)	17.2 (4.4)

BMI, Body Mass Index; M-JOA, Improvement Japanese Orthopedic Association (M-JOA) Lumbago Score Scale; SD, standard deviation; LDH, lumbar disc herniation.

**Table 2:** Comparison of M-JOA scores.

Variable	Week 2			Month 6		
	Mean (SD)		95% CI	Mean (SD)		95% CI
Group A	3.8 (2.6)		3.4~4.2	3.7 (2.2)		3.3~4.1
Group B	7.9 (3.0)		7.4~8.4	8.9 (3.1)		8.4~9.4
Group C	8.5 (2.9)		8.0~9.0	10.1 (2.9)		9.5~10.6
Comparison between the three groups						
*F* value		3.8			5.2	
*P* value		0.016			0.008	
Group A versus Group B						
*q* value		4.1			5.9	
*P* value		0.022			0.013	
Group A versus Group C						
*q* value		5.1			6.7	
*P* value		0.017			0.002	
Group C versus Group B						
*q* value		2.0			3.2	
*P* value		0.146			0.041	

Comparison between the three groups by ANOVA test. Pairwise comparison for the two groups by Student-Newman-Keuls (*q*-test). All data are intended to treat. Each group *n* = 152. SD: standard deviation; M-JOA: Improvement Japanese Orthopedic Association (M-JOA) Lumbago Score Scale; SD: standard deviation; LDH: lumbar disc herniation; Group A: Heat-sensitive moxibustion group; Group B: Conventional moxibustion group; Group C: Conventional drug plus acupuncture group.
